# Human Brain Stem Structures Respond Differentially to Noxious Heat

**DOI:** 10.3389/fnhum.2013.00530

**Published:** 2013-09-06

**Authors:** Alexander Ritter, Marcel Franz, Caroline Dietrich, Wolfgang H. R. Miltner, Thomas Weiss

**Affiliations:** ^1^Department of Biological and Clinical Psychology, Friedrich Schiller University, Jena, Germany

**Keywords:** A-delta fiber, C-fiber, second pain, pain descriptors, PAG, RVM, periaqueductal grey, rostral ventromedial medulla

## Abstract

Concerning the physiological correlates of pain, the brain stem is considered to be one core region that is activated by noxious input. In animal studies, different slopes of skin heating (SSH) with noxious heat led to activation in different columns of the midbrain periaqueductal gray (PAG). The present study aimed at finding a method for differentiating structures in PAG and other brain stem structures, which are associated with different qualities of pain in humans according to the structures that were associated with different behavioral significances to noxious thermal stimulation in animals. Brain activity was studied by functional MRI in healthy subjects in response to steep and shallow SSH with noxious heat. We found differential activation to different SSH in the PAG and the rostral ventromedial medulla (RVM). In a second experiment, we demonstrate that the different SSH were associated with different pain qualities. Our experiments provide evidence that brainstem structures, i.e., the PAG and the RVM, become differentially activated by different SSH. Therefore, different SSH can be utilized when brain stem structures are investigated and when it is aimed to activate these structures differentially. Moreover, percepts of first pain were elicited by shallow SSH whereas percepts of second pain were elicited by steep SSH. The stronger activation of these brain stem structures to SSH, eliciting percepts of second vs. first pain, might be of relevance for activating different coping strategies in response to the noxious input with the two types of SSH.

## Introduction

Nociceptive stimulation evokes activity in a number of brain structures including the brain stem. Thereby differential nociceptive stimulation in animals leads to differential activity in the brain stem. Studies in rats indicate that brain stem structures related to nociception like the periaqueductal gray (PAG) and the nucleus Raphe magnus (NRM) in the rostral ventromedial medulla (RVM), are activated differentially by different slopes of skin heating (SSH) (Lumb et al., 2002; Lu et al., 2004; Parry et al., 2008). So the dorsolateral PAG was shown to be preferentially activated in response to steep SSH while activation of the ventrolateral PAG was observed preferentially to shallow SSH (Lumb et al., 2002; Parry et al., 2008). Furthermore, Lu et al. (2004) revealed in rats that activation of the NRM (and nocifensive effects) were different for steep vs. shallow SSH.

Moreover, brain stem activity is directly associated with modulation of pain intensity. So, electrical stimulation of the PAG, one of the brain stem areas usually activated by nociceptive input, has been shown to produce analgesia (Basbaum and Fields, 1984). In animals, distinct brain stem structures have been shown to be associated with distinct behavioral and cardiovascular components of nociceptive reaction. In humans, studies already revealed the importance of brain stem structures for the modulation of pain (Bromm and Treede, 1987b; Behbehani, 1995; Bandler et al., 2000). Recently, placebo analgesia was directly associated with the activity of PAG and RVM (Eippert et al., 2009). This might be of clinical importance because a specific activation of the brain stem could be associated with a reduction of pain perception. Such a pain-modulation would be interesting especially for chronic pain patients.

Taking into account the above-mentioned activations of PAG and RVM in response to different SSH in animals, we aimed to investigate brain stem activation to two different SSH in humans. However, the paradigm used in animals is difficult to realize in human due to at least two reasons: first, nociceptive stimulation in animals was realized with temperatures up to 60°C for a longer period of time. Such stimulation would cause serious injury in humans (Lumb, 2002; Lu et al., 2004). Second, there were single heat ramps with a delay of 8 min between two stimulations in the animal experiments. This delay is too long even for a block design in functional MRI (fMRI). To our knowledge, there are no studies using different SSH in humans to investigate brain stem activation. However, there are human studies using trains of thermal stimuli with different frequencies (Price et al., 1977; Staud et al., 2007). In these studies, trains of thermal stimuli with different intervals between noxious heat stimulations were applied. Modulating frequency of heat stimulation, humans reported different pain percepts (Price et al., 1977; Price, 1988; Staud et al., 2007) that might be assigned to two distinct conceptual entities, i.e., “first pain” and “second pain.” The so called “first pain” can be clearly localized, feels pricking, and occurs fast and first after nociceptive stimulation (Bromm and Treede, 1987a; Magerl et al., 1999; Beissner et al., 2010). First pain is considered to inform the individual about the location of an injury at and within the body and about the sensory quality of the injury. The so called “second pain” can less clearly be localized. Second pain is described as dull or pressing and occurs later after nociceptive stimulation than first pain (Price, 1988; Miltner, 1989; Magerl et al., 1999; Beissner et al., 2010). The prolonged second pain is considered to pull the individuals attention to the injury and to convey information to the brain that provides the basis for pain-related affect, arousal, and behavioral responses to limit further injury and to optimize recovery. Concerning the two types of pain, it has been shown that second pain is enhanced and first pain is suppressed when moderately painful heat is presented with a frequency of greater than 0.3 Hz (Price et al., 1977; Staud et al., 2007). When painful heat is presented with frequencies below 0.17 Hz, first pain is not suppressed and no enhancement of second pain occurs (Price et al., 1977; Staud et al., 2007). This is in line with Bromm’s and Treede’s suggestion (Bromm and Treede, 1987a) that second pain is perceived when first pain is reduced and vice versa.

In humans, different SSH have not been investigated to evoke different activation in brain stem structures so far. With the first (fMRI) experiment, we aimed at finding a method to test whether noxious heat stimulations with different SSH does activate brain stem structures differentially. According to animal studies, we expect a differential activation of PAG and RVM to different SSH. With the second experiment, we tested whether the different SSH used in the fMRI environment are associated with different pain qualities as stimulation with different SSH were associated with different behavioral responses in animal studies (Lumb, 2002).

## Materials and Methods

We conducted two experiments, one inside and one outside the fMRI scanner. Both experiments used the same thermal stimulation with steep and shallow SSH. Subjects were informed about the procedure and provided written informed consent. To familiarize the participants with the experimental procedure and the stimulus types, each subject received a brief demonstration of the thermal stimulation prior to the experiment. Participants were otherwise naive about the purpose of the experiments. No subject had any history of neurological, psychiatric, or pain disorder. They were free to withdraw from the experiment at any time. The procedure was approved by the local ethics committee of the Friedrich Schiller University of Jena.

### Determination of the pain sensitivity

Thermal stimuli were applied by a fMRI-compatible Peltier thermode (Medoc Advanced Medical systems; Ramat Yishai, Israel). The thermode had a surface area of 9 cm^2^. Subjects were instructed to rate a series of thermal stimuli applied to the thenar eminence of their left hand using a modified Ellermeier scale (Ellermeier et al., 1991). This scale starts with 0 for “no pain” with an open scale with verbal description 1–10 = “just perceived,” 11–20 = “clearly perceived but not painful,” 21–30 = “very slightly painful,” 31–40 = “slightly painful,” 41–50 = “medium pain,” 51–60 = “strong pain,” 61–70 = “very strong pain.” It was explained that pain should be rated with values higher than 70 if pain becomes worse. The original Ellermeier scale has good psychophysical properties (Ellermeier et al., 1991). We included 20 additional steps at the lower end to represent non-painful perceptions. Subjects were instructed to make a judgment regarding the categories first and subsequently rate the pain intensity within the range defined by this category. The rating requested was just the discrete number of rating that was monitored for further analysis.

To determine the pain sensitivity, thermode temperature was increased to a maximal stimulation temperature of 44°C–49°C in steps of 1°C. The procedure was as follows: a starting temperature of 34°C was established. Then, one full ramp with rise and fall of 10°C (2.5°C/s) was applied providing a maximal temperature of 44°C. Subject’s intensity rating was recorded. The next starting temperature with a step of 1°C was established (up to a maximum of 39°C), followed by the next ramp with similar parameters (increase of 10°C with 2.5°C/s rise and fall). The procedure was finished at either 49°C maximal temperature or before maximal temperature of 49°C when subjects reported a rating of 51 or higher on the scale described above. This procedure allows the fitting of a stimulus-response curve presenting subjective ratings in dependence of the maximal temperature used for stimulation. It also familiarized the subjects with the kind of stimulation of the main experiments. For the succeeding main experiments, a T_hot_ was determined as the temperature where the subject reported a value of 50 on our modified Ellermeier scale. T_hot_ of all participants varied between 46.5°C and 49°C. Another maximal temperature of stimulation was used (T_warm_ = 40°C) providing ratings in the range below 20 on our scale.

### Thermal stimulation

Trains of thermal stimuli with different SSH are used for the experiments. This is an ecologically valid procedure to induce pain percepts. Thermal stimuli were applied to the thenar eminence of the right hand. Subject received two different types of heat pulse trains (steep vs. shallow SSH) applied with two different temperature levels (T_hot_ vs. T_warm_). Heat pulse trains were balanced to control for order effects.

**Figure 1:**
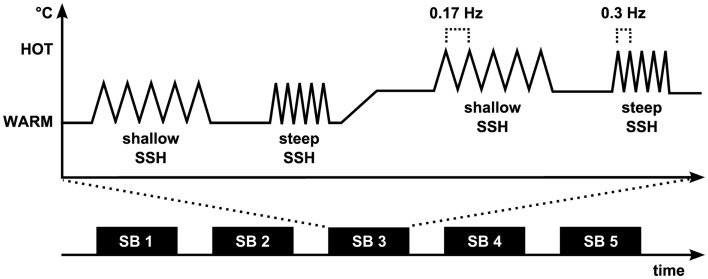
**Experimental paradigm**. The fMRI experiment (Experiment 1) consisted of five stimulation blocks (SB). Each of the four stimulation conditions was presented once during each SB. Painful heat peaks of the steep SSH stimuli were applied with a frequency of 0.3 Hz whereas painful heat peaks of the stimuli with shallow SSH were applied with a frequency of 0.17 Hz. During Experiment 2, shallow and steep SSH were applied only for the hot temperature.

A design with four conditions was used, steep SSH with T_warm_, steep SSH with T_hot_, shallow SSH with T_warm_, and shallow SSH with T_hot_ (Figure 1). The four conditions were presented in stimulation blocks. Five stimulation blocks containing one of each condition were presented throughout the whole experiment (Figure 1). Within a stimulation block, a baseline of at least 20 s (see below) was introduced between conditions (Figure 1). Each condition consisted of five heating ramps of identical type. During *WARM* conditions, temperature rose to 40°C (T_warm_), whereas in the *HOT* conditions temperatures rose to T_hot_. The baseline temperature before stimulation was set to 10°C below T_warm_/T_hot_ and rose to these target temperatures with two different slopes: *steep SSH* runs had a slope of 7.5°C/s and *shallow SSH* runs had a slope of 2.5°C/s (Figure 1). Thus, painful heat peaks of the steep SSH stimuli were applied with a frequency of 0.3 Hz, whereas painful heat peaks of the stimuli with shallow SSH were applied with a frequency of 0.17 Hz in the *HOT* conditions. There was a baseline interval between stimulation blocks of 30 s.

A 30 s time interval with a constant baseline temperature (10°C below T_warm_ in the *WARM* and 10°C below T_hot_ in the *HOT* conditions) was introduced between the steep and the shallow SSH conditions of each temperature. The baseline temperature rose over the course of 20 s from 10°C below T_warm_ to 10°C below T_hot_ for a change in stimulation from *WARM* to a succeeding *HOT* condition, or decreased from 10°C below T_hot_ to 10°C below T_warm_ for a change in the stimulation from *HOT* to a succeeding *WARM* condition, respectively (Figure 1). This temperature was kept for another 20 s before the next heating ramps began. The sample was split concerning the order of shallow and steep SSH heating to control for order effects of conditions.

#### Experiment 1

Experiment 1 investigated the activation of the brainstem by means of fMRI for the different SSHs.

Sixteen healthy, right-handed subjects (seven male, nine female, 19–28 years) volunteered in the fMRI experiment. Subjects were paid €12 for completing the experiment. Prior to the experiment, the stimulus-response function to thermal stimulation of the subjects was examined as outlined above.

### Functional image acquisition

**Figure 2:**
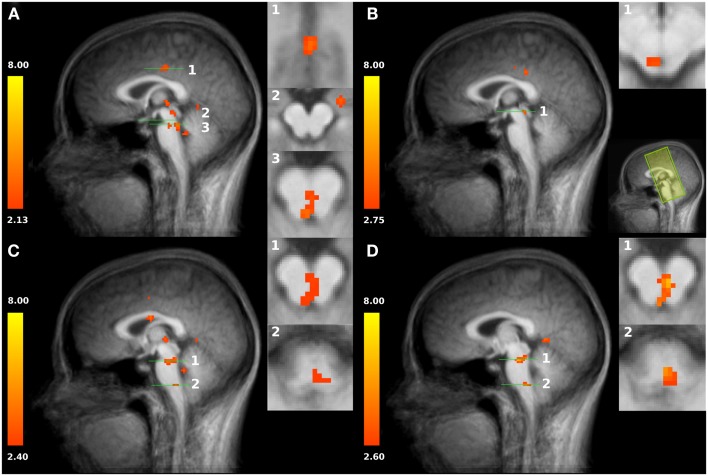
**(A)** Increased activation of both *HOT* conditions compared to baseline in the posterior cingulate cortex PCC (slice plane 1), amygdala (slice plane 2), and PAG/NRD (slice plane 3). **(B)** Increased activation to shallow SSH compared to baseline in superior part of the PAG (slice plane 1); Field of view (FOV) with coronal slices **(C)** Increased activation to steep SSH compared to baseline in inferior part of the PAG, NRD (slice plane 1), and RVM (slice plane 2). **(D)** Increased activation to steep SSH compared to shallow SSH in PAG, NRD (slice plane 1), and RVM (slice plane 2). Statistical parametric maps are overlaid on a T1 scan (neurological convention, left = left).

**Figure A1:**
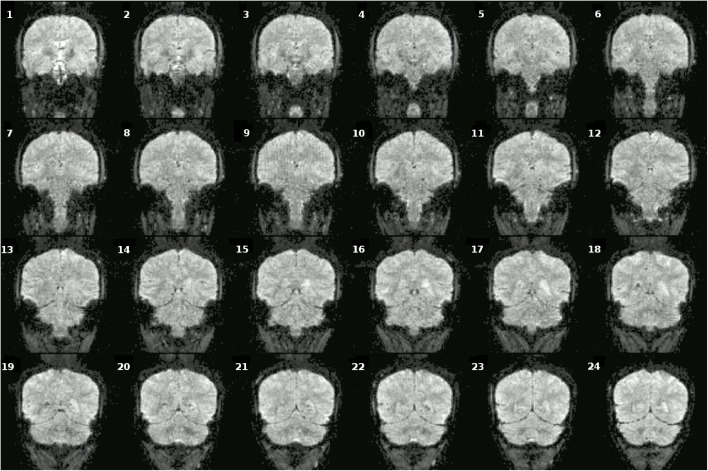
**Coronal slices (one volume) of fMRI data overlaid on a anatomical scan (neurological convention left = left)**.

Scanning was performed with a 3T magnetic resonance scanner (Tim Trio, Siemens Medical Systems, Erlangen, Germany). The experiment started with a high-resolution T1-weighted scan of the brain (192 slices, TE = 5 ms, FOV: 256 mm × 256 mm, resolution: 1 mm × 1 mm × 1 mm) for anatomical referencing and visualization. A shimming procedure preceded the succeeding functional MR scanning. The first four volumes were discarded in order to improve field homogeneity. In the experimental fMRI run, 650 volumes were acquired using a T2^∗^ weighted echo-planar sequence (TE = 75 ms, TR = 1.8 s; FOV = 192 mm × 192 mm). Each volume comprised 24 slices (2 mm thickness and 2 mm × 2 mm in-plane resolution) (see Figure A1 in Appendix) which were prescribed parallel to the brainstem. The FOV covered the *a priori*-defined region of interest which was centered around the PAG and enclosed the upper brainstem and the midbrain (Figures 2B, A1 in Appendix).

### fMRI preprocessing

**Figure A2:**
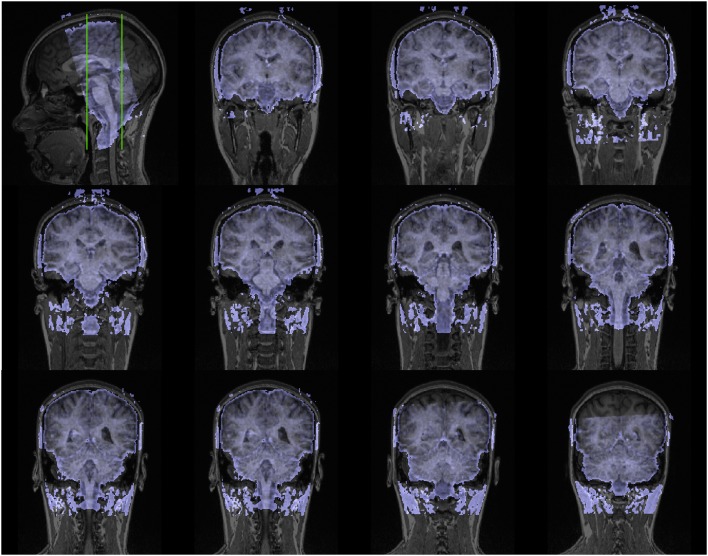
**Section (between green lines in mid – saggital view) of the functional mean BOLD – data coregistered to the anatomical scan (neurological convention left = left)**.

Preprocessing and analysis of fMRI data was performed using BrainVoyagerQX 2.1 (Brain Innovation, Maastricht, Netherlands). Primarily, all volumes were realigned to the first volume in order to minimize effects of head movements on data analysis. Further data preprocessing comprised spatial (6 mm full-width half-maximum isotropic Gaussian kernel) and temporal smoothing (high pass filter: 15 cycles per run; low pass filter: 2.8 s; linear trend removal). The anatomical and functional images were co-registered (Figure A2 in Appendix) and normalized to the Talairach space (Talairach and Tournoux, 1988).

### fMRI statistical analysis

Statistical analyses were performed by multiple linear regression of the signal time course at each voxel. The expected blood oxygen-level-dependent (BOLD) signal change for each of the four conditions (predictors) was modeled by a canonical hemodynamic response function. A random-effects General Linear Model was used to identify associated brain activity in all acquired slices. To minimize false-positive results (Straube et al., 2008) we tested whether the detected clusters survived a correction for multiple comparisons. We used the approach as implemented in Brain Voyager (Goebel et al., 2006), which is based on a 3D extension of the randomization procedure described by Forman et al. (1995). This procedure is based on the estimate of the map’s spatial smoothness and on an iterative procedure (Monte Carlo simulation) for estimating cluster-level false-positive rates. After 1000 iterations, the minimum cluster size threshold that yielded a cluster-level false-positive rate of 5% was applied to the statistical maps. Clusters reported here survived this control of multiple comparisons. For subsequent visualization of activated brain regions, the location of significantly activated regions was assessed by superimposing the results from group analysis on an averaged brain.

As the intent of this study was to characterize the changes in blood oxygen level dependent (BOLD) response to the painful stimulation, we compared the two different SSHs in the *HOT* conditions. The brain stem clusters found for this contrast constitute the basis for the analysis of further effects. The coordinates of the peak voxels were allocated to the anatomical structures with the assistance of an atlas of the human brain stem (Paxinos and Huang, 1995). For this comparison we also conducted repeated measures *t*-tests for the peak voxel of each structure. Additionally, conditions we used repeated measures ANOVAs to assess the differential effects of the four conditions.

#### Experiment 2

Experiment 2 was conducted to prove whether the different SSH are able to elicit different pain percepts. This is an important question with respect to the discussion concerning different types of afferents possibly involved during this type of stimulation.

Prior to the experiment, the stimulus-response function of the subjects was assessed analogously to Experiment 1. Stimuli with different SSH were then applied similarly to Experiment 1 with two exceptions: first, there was no *WARM* condition included. Second, the *HOT* condition was presented 10 times (5 times with steep and 5 times with shallow SSH). Directly after each *HOT* condition, subjects were requested to indicate how the stimuli of different SSHs were perceived. We used a (restricted) three-item verbal descriptor list which has been shown that it can reliably indicate whether the pain sensation evoked by the physical stimulus is the result of predominantly Aδ (first pain) or C-fiber activity (second pain) (Beissner et al., 2010). According to Beissner et al. (2010), “pricking” is an indicator for the first pain, while “pressing” or “dull” are indicators for the second pain. Thus, subjects were requested to choose the appropriate perception(s) from this list of three adjectives for the previous stimulation.

Twenty-three healthy right-handed subjects (3 male, 20 female, 19–28 years) volunteered in Experiment 2. Subjects were paid €5 for completing the experiment.

For the analysis of the data of Experiment 2, odds ratios (OR) were calculated separately for each of the three descriptors according to Beissner et al. (2010) as (A⋅D)/(B⋅C). The capital letters have the following meaning:

A: number of selections of the given descriptor for stimulations with steep SSH;
B: total number of stimulations with steep SSH minus A (i.e., the number of selections of the given descriptor for stimulations with steep SSH);
C: number of selection of the given descriptor for stimulations with shallow SSH;
D: total number of stimulations with shallow SSH minus C (i.e., number of selection of the given descriptor for stimulations with shallow SSH).

If “pricking” will be chosen more often for shallow SSH (OR < 1), then we might conclude that this stimulation preferentially activates Aδ-fibers. Accordingly, if “pressing” and/or “dull” will be chosen more often for steep SSH (OR > 1), then we might conclude that this stimulation preferentially activates C-fibers. Ninety-five percent confidence intervals, calculated as OR ± 1.96⋅(1/A + 1/B + 1/C + 1/D)^0.5^, were utilized to evaluate the significance of the respective ORs.

## Results

### Experiment 1

**Figure 3:**
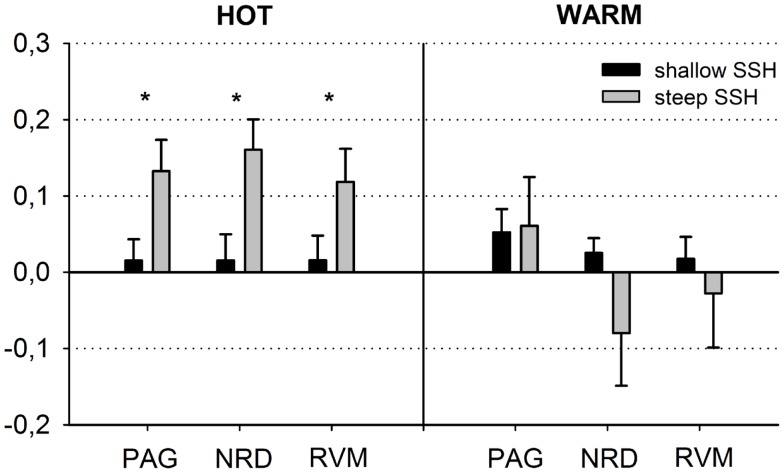
**Schematic overview of the blood oxygen level dependent (BOLD) responses in the periaqueductal gray (PAG), the nucleus Raphe dorsalis (NRD), and the rostral ventromedial medulla (RVM) (steep vs. shallow SSH for T_hot_ and T_warm_ conditions)**. The figure depicts means ± SE of parameter estimates for the peak voxel of the structures.

First, we tested whether brain stem regions show activation during the noxious thermal stimulation compared to baseline. We found activation to both SSH in the painful *HOT* conditions compared to baseline in an inferior part of the PAG, probably including nucleus Raphe dorsalis (NRD) according to (Paxinos and Huang, 1995) [*t*(15) = 3.354, *p* < 0.005, *x*, *y*, *z*: 0, −29, −20] (Figure 2A, No. 3). More specifically, stimulation with shallow SSHs (vs. baseline) led to higher activation in a more superior part of the PAG [*t*(15) = 3.24, *p* < 0.01, *x*, *y*, *z*: −3, −28, −6, Figure 2B], whereas the stimulation with the steep SSHs yielded higher activation in the PAG/NRD complex [*t*(15) = 3.77, *p* < 0.005, *x*, *y*, *z*: 1, −25, −19, Figure 2C]. Furthermore, the steep SSHs in the T_hot_ condition showed activation in a brain stem cluster that probably represents the RVM according to (Paxinos and Huang, 1995) [*t*(15) = 3.12, *p* < 0.01, *x*, *y*, *z*: 3, −33,−43, Figure 2C]. More importantly, we investigated whether these brain stem regions show differential responses under the two painful experimental conditions (*HOT*), i.e., with steep vs. shallow SSHs. This contrast revealed significantly stronger activation in the inferior part of the PAG [*t*(15) = 3.54, *p* < 0.005, 16 voxel, *x*, *y*, *z*: 1, 31, 17], NRD [*t*(15) = 4.93, *p* < 0.001, 40 voxel, *x*, *y*, *z*: 2, 25, 18], and RVM [*t*(15) = 3.82, *p* < 0.005, 16 voxel, *x*, *y*, *z*: 2, 30, 42]. No significant differences were found for the comparison between steep and shallow SSH in the *WARM* conditions (PAG [*t*(15) = 0.18, *p* > 0.1], NRD [*t*(15) = 1.57, *p* > 0.1], and RVM [*t*(15) = 0.53, *p* > 0.1]). The clusters of higher activation to steep vs. shallow SSHs are shown in Figure 2D; β-values of BOLD responses are shown in Figure 3. In contrast to the higher activation observed for steep SSHs, we did not find any statistically significant difference for the comparison shallow SSHs vs. steep SSHs for T_hot._

Repeated measures ANOVAs for main effects and interactions of all other conditions were performed for β-values of peak voxels where significant effects were found for the contrast of interest, i.e., steep vs. shallow SSH in the *HOT* condition. ANOVA revealed a main effect of temperature for the NRD cluster with stronger activation for T_hot_, a main effect of type of SSH in PAG with stronger activation for steep SSH, and an interaction for temperature × SSH in the NRD (Table 1). The significant interaction term for the NRD was investigated with a contrast analysis. There is a higher activation for steep SSH vs. shallow SSH in the *HOT* condition [*t*(15) = 4.932, *p* < 0.001], but no significant difference in activation between steep and shallow SSH in the *WARM* condition. Conversely, we found a higher activation for steep SSH with T_hot_ as compared to with T_warm_ [*t*(15) = 4,058, *p* = 0.001] whereas no difference was found for the shallow SSH between T_hot_ and T_warm_.

**Table 1:** **Main effects and interactions of parameter estimates for the factors Temperature and SSH within the brain stem**.

	Talairach *x*, *y*, *z*	Volume	Temperature	SSH	Temperature × SSH
PAG	1, −31, −17	16	*F*(1, 15) = 0.203, *p* > 0.1	*F*(1, 15) = 5.607, *p* = 0.032	*F*(1, 15) = 2.977, *p* > 0.1
NRD	−2, −25, −18	40	*F*(1, 15) = 13.303, *p* = 0.002	*F*(1, 15) = 0.326, *p* > 0.1	*F*(1, 15) = 10.631, *p* = 0.005
RVM	2, −30, −42	16	*F*(1, 15) = 3.17, *p* = 0.095	*F*(1, 15) = 0.522, *p* > 0.1	*F*(1, 15) = 3.215, *p* = 0.0903.

Second, we used our restricted field of view (remember that the acquired slices concentrated on brainstem activation and did not allow the mapping of the whole “neuromatrix of pain” (Treede et al., 1999; Tracey and Mantyh, 2007; Iannetti and Mouraux, 2010; Schweinhardt and Bushnell, 2010)) to prove our experimental manipulation. We found higher activations to both SSH in the painful T_hot_ conditions compared to baseline in the posterior cingulate cortex [PCC, *t*(15) = 3.498, *p* < 0.005, 252 voxel, *x*, *y*, *z*: 1, −19, 35; Figure 2A, No.1], in left amygdala [*t*(15) = 3.44, *p* < 0.005, 312 voxel, *x*, *y*, *z*: 21, −10, −12; Figure 2A, No. 2], and in the medial thalamus [*t*(15) = 3.19, *p* < 0.01, 40 voxel, *x*, *y*, *z*: −1, −22, 1; Figure 2]. These results indicate that our paradigm with different SSH is suitable to activate brain regions that have been found to process noxious thermal stimuli in other studies (Peyron et al., 2000).

### Experiment 2

**Figure 4:**
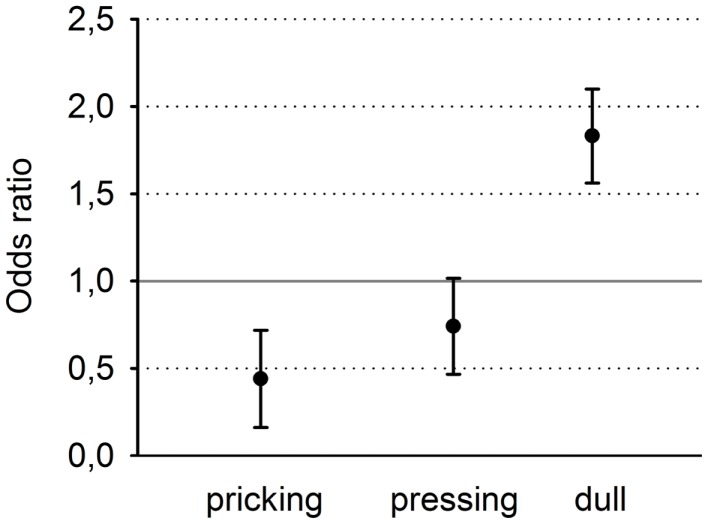
**Odds ratios and 95% confidence intervals of the three descriptors, sorted from left to right for increasing selectivity for second pain and decreasing selectivity for first pain**.

Odds ratios (and 95% confidence intervals) were calculated separately for the three descriptors (Figure 4). Clearly, the descriptor “pricking” which is associated with first pain was chosen significantly more often for the stimulation with the shallow SSH, whereas the descriptor “dull” which is associated with second pain, was chosen significantly more often for the stimulus with the steep SSH. The descriptions for “pressing” did not reach a significant discrimination between the different SSH (Figure 4).

## Discussion

Using different SSH, our primary finding is a stronger BOLD activity in response to trains of painful heat stimuli with steep SSH as compared to trains of painful heat stimuli with shallow SSH. Higher activation was found in the inferior part of the PAG, probably including the NRD according to (Paxinos and Huang, 1995), and a cluster that probably represents the RVM according to (Paxinos and Huang, 1995). We did not find any stronger activation in the brain stem for the contrast shallow vs. steep SSH. Thus, this method is able to differentially activate structures in the human brain stem. To our knowledge, this is the first time that differential activation to peripheral noxious stimulation is demonstrated in humans.

The stronger activation in the PAG/NRD complex and the RVM observed to steep vs. shallow SSH is surprising with respect to animal studies. In animal experiments, the shallow SSH, and not the steep SSH yielded more activation. Several reasons might account for this result. First, the maximal temperatures of stimulation differed between the present experiment (49°C) and the animal studies [e.g., 55°C (Lumb, 2002)]. It is well known that the characteristics of nociceptors differ in their response behavior for this temperature range (Behbehani, 1995). Second, the differences might be due to our spatial resolution. The resolution of brain scans with 2 mm × 2 mm × 2 mm in the present study might not have been sufficiently high to detect activations in different columns of the PAG. Third and probably most important, single ramps with shallow SSH were employed in the animal experiments. Such stimulation was shown to preferentially activate C-fibers, whereas single steep SSH are able to preferentially excite Aδ-fibers (Lumb, 2002; Lumb et al., 2002). In contrast to the animal experiments, a train of five succeeding heating ramps without gaps was employed in our study. Thereby, the steep SSH resulted in a frequency of heat peaks of 0.3 Hz. A stimulus frequency of 0.3 Hz is known to produce the phenomenon of temporal summation of second pain, i.e., TSSP (Price et al., 1977; Herrero et al., 2000). TSSP is considered to result from C-fiber evoked responses in dorsal horn neurons, termed “windup” (Herrero et al., 2000; Sarlani and Greenspan, 2005). Thus, the involved fibers activated by the steep SSH in animal studies are Aδ-fibers while the activation with repeated steep SSH in our experiment might preferentially involve C-fibers. Following this interpretation, the activation of the brain stem by steep SSH of stimulus trains in our study has to be compared to the single shallow SSH in the animal experiments. Considering this, both experiments yielded similar results.

The results of Experiment 2 are of crucial importance for the latter consideration. It investigated the quality of pain percepts that are elicited by different SSHs in humans. Indeed, we found evidence that the steep SSH was associated with the percept “dull” whereas the shallow SSH was associated with the percept “pricking.” In accordance with Beissner et al. (2010), these descriptors distinguish best between first pain and second pain. Thus, steep SSH might be associated with a predominant activation of C-fibers while the shallow SSH might be associated with a predominant activation of Aδ-fibers. This interpretation is in line with other studies using trains of painful thermal stimuli that were associated with different pain percepts (Price et al., 1977; Staud et al., 2007). Characteristics of first pain were associated with a frequency of 0.17 Hz between the painful heat peaks whereas characteristics of second pain were associated with a frequency of 0.3 Hz between the painful heat peaks (Price et al., 1977; Staud et al., 2007). However, it should be mentioned that heat is perceived as painful in humans only at temperatures above 43°C (Julius and Basbaum, 2001). Thus, just the top of each heating ramp can be considered as a painful heat peak. In the present study, the painful heat peaks of the steep SSH stimuli were applied with a frequency of 0.3 Hz whereas heat peaks of the stimuli with shallow SSH were applied with a frequency of 0.17 Hz so that these ramps fulfill the frequency criterion for painful stimulation. Taking together the two experiments, we suggest that the different SSH probably activate different types of peripheral input resulting in different pain percepts (first vs. second pain).

We found activation of the PAG and the RVM to stimulation with noxious heat to both types of SSH. Several brain imaging studies have observed activations in brainstem structures to nociceptive stimulation (Apkarian et al., 2005; Tracey and Mantyh, 2007; Eippert et al., 2009; Schweinhardt and Bushnell, 2010). Brainstem modulation of neuronal activity in the spinal cord has been reported since more than a century ago (Bernard, 1858) and is thought being involved in top-down control of pain. In particular, the midline PAG integrates input from the spinal cord, cerebral cortex, and numerous other brainstem nuclei (Apkarian et al., 2005; Eippert et al., 2009). Its stimulation in humans was shown to result in antinociception and analgesia (Hosobuchi et al., 1977). In contrast, its lesion might result in chronic pain (Basbaum and Fields, 1984). Our result of an increased activity in the inferior part of the PAG in response to steep as compared to shallow SSH might, therefore, be an important result. Based on the animal studies mentioned above, this higher activation might indicate that the steep SSH stimulation, but not the shallow SSH stimulation might trigger the nocifensive part of PAG. Moreover, the hypothesis that this type of activation might result in an activation of nocifensive reaction might be tested and, if true, possibly be used for chronic pain patients.

We found a higher activation within the inferior part of the PAG to stimulation with steep as compared to shallow SSH. This result is in line with previous studies in animals. Animal studies have found different activation patterns within distinct columns of the PAG to preferential C- and Aδ-fiber stimulation (Lumb, 2002; Lumb et al., 2002; Parry et al., 2008). It has also been proposed that the different columns of the PAG not only differ with respect to the influence of the ongoing nociceptive information processing as outlined above, but also mediate different coping strategies (Lumb et al., 2002). Preferential Aδ-fiber stimulation is associated with activation of dorsolateral and lateral columns of the PAG which in turn result in active coping strategies (Lumb et al., 2002). The activation of these columns evokes sympathetic excitation. Passive coping strategies are closely linked to the ventrolateral columns of the PAG activated by preferential C-fiber stimulation. The activity of the ventrolateral PAG is associated with sympathoinhibition (Lumb et al., 2002). Correspondingly, the PAG mediates differential control of spinal nociception as part of a defensive response or as withdrawal. It has been established to carry out integrative functions for cardiovascular and respiratory regulation, for sensory modulation, and for different motor behaviors (Clement et al., 2000; Morgan and Carrive, 2001; Subramanian et al., 2008; Heinricher et al., 2009). Reflecting these results on our data, the findings of this study provide a hint that stimulation with steep SSH but not shallow SSH might engage nocifensive mechanisms as shallow SSH do not seem to influence the PAG in the same way as steep SSH.

Although our main focus in this study was the PAG, we found activations in two neighboring and functionally related areas, i.e., in NRD and RVM. The NRD is embedded in the ventromedial part of the PAG (Mantyh, 1982) and was shown to modulate responses caused by noxious stimulation of the spinal dorsal horn neurons by its descending projections (Yu et al., 1988). In addition, PAG and NRD project to the spinal cord indirectly via the RVM, which is situated centrally around the pontomedullary junction. It includes the NRM and the adjacent reticular formation. It is known to project diffusely to dorsal horn laminae, including superficial layers and deep dorsal horn structures (Fields and Heinricher, 1985). Similar to the PAG, the RVM has a dual role in pain control: it is as well able to inhibit and to facilitate nociceptive input and can thus be considered as the output of the midline pain-modulation system. Profound analgesia can be produced by stimulating the NRM which is due to a decrease in responsiveness of spinothalamic dorsal horn neurons to input from peripheral nociceptors (Besson and Chaouch, 1987). Alternatively, analgesia evoked by stimulation of the ventral sites of the PAG can be blocked by lesion of the RVM (Behbehani and Fields, 1979; Prieto et al., 1983). In the light of these data, activations found in the present study might mirror the descending pathway to the dorsal horn. These observations parallel the results of the present study that stronger activation in RVM can be observed in response to steep SSH.

We found stronger activation in response to steep SSH stimulation both in parts of the brain stem as well as in some other structures in the field of view, i.e., the PCC and the amygdala. As argued above, we suggest that stimulation with steep SSH might preferentially activate C-fiber input. In this sense, the fMRI results of our study are in line with previous studies that also found stronger activation to selective C-fiber stimulation compared to Aδ-fiber stimulation. Stronger activations have been reported in structures associated with the affective processing of nociceptive information (i.e., ACC (Qiu et al., 2006); anterior insula (Weiss et al., 2008)). Similar to the present study these authors (Qiu et al., 2006; Weiss et al., 2008) also did not find any stronger activation to selective Aδ-fiber stimulation when comparing it with selective C-fiber stimulation. This might be another hint to the correctness of our suggestion concerning preferential C-fiber activation by steep SSH stimulation.

Several limitations of our study have to be considered that might influence future research. First, the conditions steep and shallow SSH were determined by the different slopes of heating. Different slopes affect the frequency of the painful heat peaks that are essential for the paradigm. There are two possibilities to proceed further, with the same number of stimuli within a condition (i.e., 5× up and down) or the same duration within a condition, but a different number of stimuli within a condition. We decided to use the same number of stimuli to have the same number of painful events within a condition. However, this leads to different durations of stimulation between the two SSHs used. Future studies might explore the effects of the two types of SSHs using the same length but unequal number of painful events within a condition.

Second, the energy transmitted to the skin depends on the frequency and duration of stimulation within a condition. In our study, the transmitted energy (area under the curve) was higher in the shallow SSH condition. Future studies might utilize the same amount of transmitted energy. However, different slopes of heating will then request different durations of baseline between ramps. These segments in turn might rise additional percepts in difference to the heat stimulation that might influence the results. However, it should be mentioned that heat pain receptors start firing at about 43°C (Julius and Basbaum, 2001) so that it is quite difficult to produce ramps that have the same amount of energy in the painful range; moreover, it seems to be impossible to produce ramps with different SSH that have the same energy both in the noxious as well as in the innocuous temperature range. Taking this consideration into account, the difference in transferred energy above the temperature threshold of 43°C is smaller as compared to differences in transferred energy for the whole heating ramps.

Third (as mentioned earlier), the resolution of 2 mm × 2 mm × 2 mm might not be sufficient to detect further differentiation within the brain stem, especially within the PAG. Possibly, scanning with higher field strengths might identify a columnar organization of the human PAG.

In summary, we found stronger activation in the inferior part of the PAG and in the RVM in response to painful stimulation with steep SSH. These observations provide first evidence for selective activation of the midbrain structures PAG and RVM in the human brainstem by different SSH. Therefore, this stimulation can be used when human brainstem structures are in the focus of interest during nociception. The specific activation of the midbrain to steep SSH seems to be associated with the specific perception of second pain and might possibly be related to passive coping strategies.

## Conflict of Interest Statement

The authors declare that the research was conducted in the absence of any commercial or financial relationships that could be construed as a potential conflict of interest.

## References

[B1] Apkarian A. V. Bushnell M. C. Treede R. D. Zubieta J. K. (2005). Human brain mechanisms of pain perception and regulation in health and disease. Eur. J. Pain 9, 463–484 10.1016/j.ejpain.2004.11.001 15979027

[B2] Bandler R. Keay K. A. Floyd N. Price J. (2000). Central circuits mediating patterned autonomic activity during active vs. passive emotional coping. Brain Res. Bull. 53, 95–104 10.1016/S0361-9230(00)00313-0 11033213

[B3] Basbaum A. I. Fields H. L. (1984). Endogenous pain control systems: brainstem spinal pathways and endorphin circuitry. Annu. Rev. Neurosci. 7, 309–338 10.1146/annurev.ne.07.030184.001521 6143527

[B4] Behbehani M. M. (1995). Functional characteristics of the midbrain periaqueductal gray. Prog. Neurobiol. 46, 575–605 10.1016/0301-0082(95)00009-K 8545545

[B5] Behbehani M. M. Fields H. L. (1979). Evidence that an excitatory connection between the periaqueductal gray and nucleus raphe magnus mediates stimulation produced analgesia. Brain Res. 170, 85–93 10.1016/0006-8993(79)90942-9 223721

[B6] Beissner F. Brandau A. Henke C. Felden L. Baumgartner U. Treede R. D. (2010). Quick discrimination of A(delta) and C fiber mediated pain based on three verbal descriptors. PLoS ONE 5:e12944 10.1371/journal.pone.0012944 20886070 PMC2944851

[B7] Bernard C. (1858). Leçons sur la physiologie et la pathologie du système nerveux. Paris: J.-B. Baillière et fils

[B8] Besson J. M. Chaouch A. (1987). Peripheral and spinal mechanisms of nociception. Physiol. Rev. 67, 67–186 3543978 10.1152/physrev.1987.67.1.67

[B9] Bromm B. Treede R. D. (1987a). Human cerebral potentials-evoked by Co2-laser stimuli causing pain. Exp. Brain Res. 67, 153–162 10.1007/BF00269463 3622675

[B10] Bromm B. Treede R. D. (1987b). Pain related cerebral potentials: late and ultralate components. Int. J. Neurosci. 33, 15–23 10.3109/00207458708985926 3610490

[B11] Clement C. I. Keay K. A. Podzebenko K. Gordon B. D. Bandler R. (2000). Spinal sources of noxious visceral and noxious deep somatic afferent drive onto the ventrolateral periaqueductal gray of the rat. J. Comp. Neurol. 425, 323–344 10.1002/1096-9861(20000925)425:3<323::AID-CNE1>3.0.CO;2-Z 10972936

[B12] Eippert F. Bingel U. Schoell E. D. Yacubian J. Klinger R. Lorenz J. (2009). Activation of the opioidergic descending pain control system underlies placebo analgesia. Neuron 63, 533–543 10.1016/j.neuron.2009.07.014 19709634

[B13] Ellermeier W. Westphal W. Heidenfelder M. (1991). On the “absoluteness” of category and magnitude scales of pain. Percept. Psychophys. 49, 159–166 10.3758/BF03205035 2017352

[B14] Fields H. L. Heinricher M. M. (1985). Anatomy and physiology of a nociceptive modulatory system. Philos. Trans. R. Soc. Lond. B Biol. Sci. 308, 361–374 10.1098/rstb.1985.0037 2858889

[B15] Forman S. D. Cohen J. D. Fitzgerald M. Eddy W. F. Mintun M. A. Noll D. C. (1995). Improved assessment of significant activation in functional magnetic resonance imaging (fMRI): use of a cluster-size threshold. Magn. Reson. Med. 33, 636–647 10.1002/mrm.1910330508 7596267

[B16] Goebel R. Esposito F. Formisano E. (2006). Analysis of functional image analysis contest (FIAC) data with brainvoyager QX: from single-subject to cortically aligned group general linear model analysis and self-organizing group independent component analysis. Hum. Brain Mapp. 27, 392–401 10.1002/hbm.20249 16596654 PMC6871277

[B17] Heinricher M. M. Tavares I. Leith J. L. Lumb B. M. (2009). Descending control of nociception: specificity, recruitment and plasticity. Brain Res. Rev. 60, 214–225 10.1016/j.brainresrev.2008.12.009 19146877 PMC2894733

[B18] Herrero J. F. Laird J. M. Lopez-Garcia J. A. (2000). Wind-up of spinal cord neurones and pain sensation: much ado about something? Prog. Neurobiol. 61, 169–203 10.1016/S0301-0082(99)00051-9 10704997

[B19] Hosobuchi Y. Adams J. E. Linchitz R. (1977). Pain relief by electrical stimulation of the central gray matter in humans and its reversal by naloxone. Science 197, 183–186 10.1126/science.301658 301658

[B20] Iannetti G. D. Mouraux A. (2010). From the neuromatrix to the pain matrix (and back). Exp. Brain Res. 205, 1–12 10.1007/s00221-010-2340-1 20607220

[B21] Julius D. Basbaum A. I. (2001). Molecular mechanisms of nociception. Nature 413, 203–210 10.1038/35093019 11557989

[B22] Lu Y. Sweitzer S. M. Laurito C. E. Yeomans D. C. (2004). Differential opioid inhibition of C- and A delta-fiber mediated thermonociception after stimulation of the nucleus raphe magnus. Anesth. Analg. 98, 414–419 10.1213/01.ANE.0000094334.12027.06 14742380

[B23] Lumb B. M. (2002). Inescapable and escapable pain is represented in distinct hypothalamic-midbrain circuits: specific roles for Adelta- and C-nociceptors. Exp. Physiol. 87, 281–286 10.1113/eph8702356 11856975

[B24] Lumb B. M. Parry D. M. Semenenko F. M. McMullan S. Simpson D. A. (2002). C-nociceptor activation of hypothalamic neurones and the columnar organisation of their projections to the periaqueductal grey in the rat. Exp. Physiol. 87, 123–128 10.1113/eph8702348 11856957

[B25] Magerl W. Ali Z. Ellrich J. Meyer R. A. Treede R. D. (1999). C- and A delta-fiber components of heat-evoked cerebral potentials in healthy human subjects. Pain 82, 127–137 10.1016/S0304-3959(99)00061-5 10467918

[B26] Mantyh P. W. (1982). The midbrain periaqueductal gray in the rat, cat, and monkey – a Nissl, Weil, and Golgi analysis. J. Comp. Neurol. 204, 349–363 10.1002/cne.902040406 6174554

[B27] Miltner W. H. R. (1989). Ereigniskorrelierte Potentiale in der Schmerzmessung und Schmerzkontrolle. Tübingen: Habilitationsschrift, Eberhard-Karls-Universität

[B28] Morgan M. M. Carrive P. (2001). Activation of the ventrolateral periaqueductal gray reduces locomotion but not mean arterial pressure in awake, freely moving rats. Neuroscience 102, 905–910 10.1016/S0306-4522(00)00513-3 11182252

[B29] Parry D. M. Macmillan F. M. Koutsikou S. McMullan S. Lumb B. M. (2008). Separation of A- versus C-nociceptive inputs into spinal-brainstem circuits. Neuroscience 152, 1076–1085 10.1016/j.neuroscience.2008.01.018 18328632

[B30] Paxinos G. Huang X. (1995). Atlas of the Human Brain Stem. San Diego: Academic Press

[B31] Peyron R. Laurent B. Garcia-Larrea L. (2000). Functional imaging of brain responses to pain. A review and meta-analysis (2000). Neurophysiol. Clin. 30, 263–288 10.1016/S0987-7053(00)00227-6 11126640

[B32] Price D. D. (1988). Psychological and Neural Mechanisms of Pain. New York: Raven Press

[B33] Price D. D. Hu J. W. Dubner R. Gracely R. H. (1977). Peripheral suppression of first pain and central summation of second pain evoked by noxious heat pulses. Pain 3, 57–68 10.1016/0304-3959(77)90035-5 876667

[B34] Prieto G. J. Cannon J. T. Liebeskind J. C. (1983). N raphe magnus lesions disrupt stimulation-produced analgesia from ventral but not dorsal midbrain areas in the rat. Brain Res. 261, 53–57 10.1016/0006-8993(83)91282-9 6301628

[B35] Qiu Y. Noguchi Y. Honda M. Nakata H. Tamura Y. Tanaka S. (2006). Brain processing of the signals ascending through unmyelinated C fibers in humans: an event-related functional magnetic resonance imaging study. Cereb. Cortex 16, 1289–1295 10.1093/cercor/bhj071 16280463

[B36] Sarlani E. Greenspan J. D. (2005). Why look in the brain for answers to temporomandibular disorder pain? Cells Tissues Organs 180, 69–75 10.1159/000086200 16088135

[B37] Schweinhardt P. Bushnell M. C. (2010). Pain imaging in health and disease – how far have we come? J. Clin. Invest. 120, 3788–3797 10.1172/JCI43498 21041961 PMC2964988

[B38] Staud R. Craggs J. G. Robinson M. E. Perlstein W. M. Price D. D. (2007). Brain activity related to temporal summation of C-fiber evoked pain. Pain 129, 130–142 10.1016/j.pain.2006.10.010 17156923 PMC1997296

[B39] Straube T. Pohlack S. Mentzel H. J. Miltner W. H. (2008). Differential amygdala activation to negative and positive emotional pictures during an indirect task. Behav. Brain Res. 191, 285–288 10.1016/j.bbr.2008.03.040 18466987

[B40] Subramanian H. H. Balnave R. J. Holstege G. (2008). The midbrain periaqueductal gray control of respiration. J. Neurosci. 28, 12274–12283 10.1523/JNEUROSCI.4168-08.2008 19020021 PMC6671706

[B41] Talairach J. Tournoux P. (1988). Coplanar Stereotaxic Atlas of the Human Brain. Stuttgart: Thieme

[B42] Tracey I. Mantyh P. W. (2007). The cerebral signature and its modulation for pain perception. Neuron 55, 377–391 10.1016/j.neuron.2007.07.012 17678852

[B43] Treede R. D. Kenshalo D. R. Gracely R. H. Jones A. K. P. (1999). The cortical representation of pain. Pain 79, 105–111 10.1016/S0304-3959(98)00184-5 10068155

[B44] Weiss T. Straube T. Boettcher J. Hecht H. Spohn D. Miltner W. H. (2008). Brain activation upon selective stimulation of cutaneous C- and Adelta-fibers. Neuroimage 41, 1372–1381 10.1016/j.neuroimage.2008.03.047 18499480

[B45] Yu G. D. Guo S. Y. Zhang H. Q. Yin Q. Z. (1988). [Effect of dorsal raphe nucleus stimulation on nociceptive response of dorsal horn neurons and efferent pathway analysis in rats]. Sheng Li Xue Bao 40, 231–239 3187557

